# Warming in the Maternal Environment Alters Seed Performance and Genetic Diversity of *Stylosanthes capitata*, a Tropical Legume Forage

**DOI:** 10.3390/genes16080913

**Published:** 2025-07-30

**Authors:** Priscila Marlys Sá Rivas, Fernando Bonifácio-Anacleto, Ivan Schuster, Carlos Alberto Martinez, Ana Lilia Alzate-Marin

**Affiliations:** 1 Plant Genetics Laboratory, Department of Genetics, Faculty of Medicine of Ribeirão Preto (FMRP-USP/RP), University of São Paulo, Ribeirão Preto 14049-900, SP, Brazil; priprisa@gmail.com (P.M.S.R.); bonifacioanacleto@usp.br (F.B.-A.); 2 Genetics Graduate Program, Department of Genetics, Faculty of Medicine of Ribeirão Preto (FMRP-USP/RP), University of São Paulo, Av. Bandeirantes 3900, Ribeirão Preto 14049-900, SP, Brazil; 3 Longping High-Tech, SP-330, km 296, Cravinhos 14140-000, SP, Brazil; ivanbio7@gmail.com; 4 Department of Biology, Ribeirão Preto School of Philosophy, Science and Literature (FFCLRP-USP/RP), University of São Paulo, Av. Bandeirantes 3900, Ribeirão Preto 14040-901, SP, Brazil

**Keywords:** climatic change, food security, seed quality, germination, genetic diversity, tropical forage legumes

## Abstract

**Background/Objectives**: Global warming and rising CO_2_ concentrations pose significant challenges to plant systems. Amid these pressures, this study contributes to understanding how tropical species respond by simultaneously evaluating reproductive and genetic traits. It specifically investigates the effects of maternal exposure to warming and elevated CO_2_ on progeny physiology, genetic diversity, and population structure in *Stylosanthes capitata*, a resilient forage legume native to Brazil. **Methods**: Maternal plants were cultivated under controlled treatments, including ambient conditions (control), elevated CO_2_ at 600 ppm (eCO_2_), elevated temperature at +2 °C (eTE), and their combined exposure (eTEeCO_2_), within a Trop-T-FACE field facility (Temperature Free-Air Controlled Enhancement and Free-Air Carbon Dioxide Enrichment). Seed traits (seeds per inflorescence, hundred-seed mass, abortion, non-viable seeds, coat color, germination at 32, 40, 71 weeks) and abnormal seedling rates were quantified. Genetic diversity metrics included the average (A) and effective (Ae) number of alleles, observed (Ho) and expected (He) heterozygosity, and inbreeding coefficient (*Fis*). Population structure was assessed using Principal Coordinates Analysis (PCoA), Analysis of Molecular Variance (AMOVA), number of migrants per generation (Nm), and genetic differentiation index (*Fst*). Two- and three-way Analysis of Variance (ANOVA) were used to evaluate factor effects. **Results**: Compared to control conditions, warming increased seeds per inflorescence (+46%), reduced abortion (−42.9%), non-viable seeds (−57%), and altered coat color. The germination speed index (GSI +23.5%) and germination rate (Gr +11%) improved with warming; combined treatments decreased germination time (GT −9.6%). Storage preserved germination traits, with warming enhancing performance over time and reducing abnormal seedlings (−54.5%). Conversely, elevated CO_2_ shortened GSI in late stages, impairing germination efficiency. Warming reduced Ae (−35%), He (−20%), and raised *Fis* (maternal 0.50, progeny 0.58), consistent with the species’ mixed mating system; A and Ho were unaffected. Allele frequency shifts suggested selective pressure under eTE. Warming induced slight structure in PCoA, and AMOVA detected 1% (maternal) and 9% (progeny) variation. *Fst* = 0.06 and Nm = 3.8 imply environmental influence without isolation. **Conclusions**: Warming significantly shapes seed quality, reproductive success, and genetic diversity in *S. capitata*. Improved reproduction and germination suggest adaptive advantages, but higher inbreeding and reduced diversity may constrain long-term resilience. The findings underscore the need for genetic monitoring and broader genetic bases in cultivars confronting environmental stressors.

## 1. Introduction

### 1.1. Global Climate Change and Agricultural Implications

Under the lowest greenhouse gas emissions scenario (SSP1-1.9), the global temperature is projected to rise by approximately 1.5–2 °C by 2050 [1]. Notably, 2024 recorded the highest global temperature since record-keeping began in 1850 [2]. Projections also suggest that atmospheric carbon dioxide (CO_2_) concentrations could reach nearly 600 ppm by mid-century, potentially exceeding 800 ppm by the end of this century [1]. With a projected global population of 9 billion by 2050, these increases in temperature and CO_2_ levels will create significant challenges for agricultural systems worldwide, driven by the rapidly growing demand for food [3]. Tropical regions, in particular, are at heightened risk, as global food security and sustainability are threatened under these conditions [4,5].

Crops around the world are expected to experience significant changes due to the global climate system. Many species are particularly sensitive to environmental fluctuations, especially during critical stages of their life cycles, such as flowering, pollination, fertilization, seed development, and germination [6,7]. In tropical regions, these changes could have a major impact on the agriculture and livestock industries [1], particularly affecting legume crops like *Stylosanthes capitata*.

### 1.2. Environmental Effects on Seed Development and Physiology

Parental environmental cues during seed development and maturation significantly impact seed biochemical composition, morphology, dormancy, dispersal, and essential quality traits such as mass, viability, germination, and vigor, across both population and community levels [8,9]. These effects are mediated through multiple mechanisms, including changes in the quantity or quality of resources allocated to seeds, modifications in seed coat composition or structure, variations in seed abortion rates, and fluctuations in hormone and enzyme levels [10,11]. Understanding these mechanisms is crucial for evaluating how tropical legumes respond to climate change, particularly in species such as *S. capitata*. Warming and elevated CO_2_ have been shown to alter seed quality parameters in legumes [12], including seed mass, germination, and vigor. Warming may reduce seed mass due to accelerated development and a shortened seed-filling period; however, this does not necessarily impair germination or vigor. In contrast, high-temperature stress occurring before seeds reach physiological maturity can inhibit germination by limiting the assimilate supply required for synthesizing essential storage compounds [12]. Under conditions of elevated CO_2_, harvest rates—the ratio of collected grains to total dry matter—might decrease because plants may have a limited capacity to efficiently utilize the increased photoassimilates [13]. On the other hand, elevated CO_2_ has also been associated with an increase in seed mass for legumes [12]. However, seed traits such as quantity, size, and overall quality show variable responses to elevated CO_2_, depending on the functional group and specific plant species [14]. While studies have extensively examined legumes such as peas [15] and soybeans [16] regarding quality seed parameters, tropical species like *S. capitata* remain underexplored, highlighting the necessity for further research on their responses to warming and elevated CO_2_ conditions.

### 1.3. Research Gaps and Agronomic Importance of the Tropical Legume S. capitata

Despite the increasing research on the effects of global warming and elevated atmospheric CO_2_ levels, there is a lack of studies specifically examining their direct impacts on seed quality, germination, and genetic diversity in tropical legumes [12,17]. This gap underscores the need for focused research on economically significant species, such as *S. capitata*. As a valuable tropical legume, *S. capitata* is both ecologically and economically significant for studying the effects of environmental conditions on seed traits. In Brazil, this species is commonly cultivated as animal feed in grazing systems where it is intercropped with grasses. It is particularly valued for its protein-rich leaves and its ability to thrive in acidic, low-fertility soils. Alongside *Stylosanthes macrocephala*, it forms a key component of the ‘Estilosantes Campo Grande’ cultivar, which is cultivated over approximately 150,000 hectares in Brazil and is mainly grown in arid biomes [18,19,20,21]. However, wild populations of both *Stylosanthes* species are increasingly threatened by reduction and displacement due to escalating environmental pressures from climate change, highlighting the urgent need for conservation efforts (Bonifácio-Anacleto et al., 2024 [22]).

Previous studies on maternal plants of *S. capitata* subjected to warming and elevated CO_2_ levels have revealed significant reproductive impacts. These include increased flower production, enhanced visitation by pollinators such as *Apis mellifera* and the native *Paratrigona lineata*, and earlier blooming times (Alzate-Marin et al., 2021 [6]). Additionally, *S. capitata*-treated plants exhibited pollen anomalies and reduced pollen viability, which were also observed, though to a lesser extent, in control treatments (Alzate-Marin et al., 2021b [23]). Analyses of crossbreeding rates showed similar results across all treatments, which may be linked to the geitonogamy behavior of the pollinators [6]. Nevertheless, pollinator activity remains essential for efficient pollen transfer, directly contributing to reproductive success, crop quality, yield, and sustained productivity. Complementary findings by (Maluf et al., 2022 [24]) and (Bonifácio-Anacleto et al., 2024 [25]), derived from experiments conducted under warming and water deficit conditions, showed that warming increased sugar concentration in *S. capitata* nectar, enhanced plant–flower visitor interactions, and accelerated germination performance in derived seeds.

### 1.4. Objectives and Hypotheses

This study builds on previous research by extending the investigation beyond reproductive traits to examine how maternal exposure to elevated temperature (+2 °C) and CO_2_ (600 ppm) influences progeny-level physiological traits, genetic diversity, and population structure in *S. capitata*. We will evaluate progeny under laboratory conditions by measuring seed number per inflorescence, hundred-seed mass, abortion and non-viable seeds, seed coat color, germination at 32, 40, and 71 weeks, seedling vigor, as well as genetic diversity and population structure. Given that warming enhances flower production and increases pollinator visitation in maternal plants [6,24], we hypothesize that it will also boost seed production per inflorescence and influence parameters of genetic diversity and population structure. Additionally, based on observations of pollen anomalies and viability [23], we expect abortion rates to remain consistent across all treatments and controls. Lastly, based on previous findings [12,13,25], we propose that both warming and elevated CO_2_ levels will alter additional seed quality traits, including seed mass, coat color, and germination dynamics.

## 2. Materials and Methods

### 2.1. Species Description

**Figure 1:**
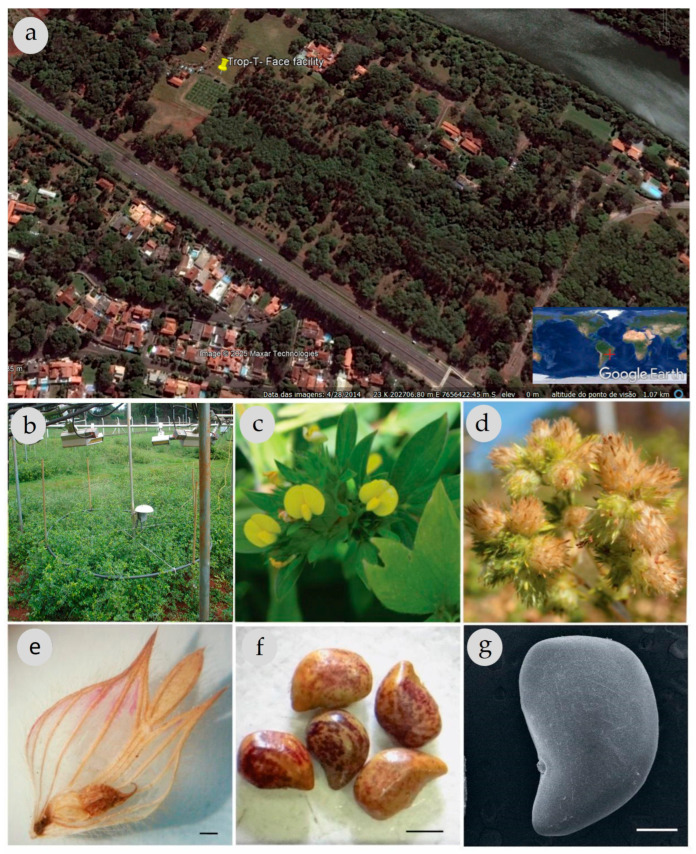
(**a**) Trop-T-FACE facility of the University of São Paulo, campus of Ribeirão Preto, SP, Brazil (Source: Historical image in Google Earth Pro. Accessed on 14 June 2025). (**b**) Detail of the infrared lamps and CO_2_ ring in the *Stylosanthes capitata* field experiment. (**c**) Papilionate flowers with yellow corollas and inflorescences. (**d**) Mature plants of *S. capitata* in the field (harvest stage). (**e**) Pods fixed to the bract extracted from mature inflorescences (4× magnification). (**f**) *S. capitata* healthy seeds extracted from pods. (**g**) scanning electron microscopy of *S. capitata* seed. Scale bars: (**e**,**f**): 1 mm, (**g**): 500 µm.

*S. capitata* Vogel is native to Brazil and Venezuela, primarily found in savanna ecosystems that receive annual precipitation ranging from 900 to 1800 mm and experience 4 to 6 dry months [18,21]. The plant produces yellow flowers with typical papilionaceous corollas (8.5 to 14.5 mm) and keels appear on terminal inflorescences (Figure 1c,d). Its seeds are broad-oblong, elliptic, or ovoid in shape, measuring 2–3 mm by 1–2 mm, and exhibit various staining patterns ([26]; see Figure 1e–g). The species typically grows to a height of 50 to 120 cm, exhibiting a sub-shrub habit (see Figure 1b,c). The stems are cylindrical and minimally woody, featuring noticeable trichomes and slender branches. The leaves are trifoliolate, with straight ribbing and a hairy texture on both surfaces.

### 2.2. Treatments

The experiment included two main phases: field treatments and laboratory analyses.

#### 2.2.1. Field Treatments in Maternal Plants

*S. capitata* commercial seeds were planted in a total of 16 plots within the experimental area using the Trop-T-FACE that combines a Temperature Free-Air Controlled Enhancement (T-FACE) and Free-Air Carbon Dioxide Enrichment (FACE) facilities, as described by (Kimball et al., 2008 [27]) and (Miglietta et al., 2001 [28]). These facilities are located at the University of São Paulo, Ribeirão Preto campus, Brazil (23K 202706.80 7656422.45) (Figure 1a). The experimental treatments, which were implemented during the growth and reproductive periods, were arranged in a randomized block design with four replicates each. The treatments included: (1) control—ambient temperature and ambient CO_2_ levels (aTEaCO_2_); (2) elevated CO_2_ levels (~600 ppm) with ambient temperature (aTEeCO_2_); (3) elevated temperature (+2 °C) with ambient CO_2_ levels (eTEaCO_2_); and (4) a combination of both elevated CO_2_ and temperature (eTEeCO_2_).

In the T-FACE system, for warming plots, six infrared lamps (IR model FTE-1000, 1000 W, 240 V) were utilized to maintain the canopy temperature at +2 °C above ambient levels (Figure 1b). Temperature control was achieved using a proportional-integrative-derivative (PID) algorithm installed in a CR1000 datalogger, which was connected to AM25T multiplexors (Campbell Scientific, Logan, UT, USA). The control system monitored the canopy temperature in the aTEaCO_2_ plots and adjusted it to be 2 °C higher than the ambient temperature in the warmed plots (eTEaCO_2_, eTEeCO_2_).

The elevated CO_2_ (FACE) system employed a 12-ton cryogenic tank filled with liquid CO_2_ at −180 °C, coupled with a vaporizer to convert it into gaseous CO_2_, which was then distributed in the field through 2 m laser-perforated diameter rings (Figure 1b). Additionally, non-perforated rings were installed in the aTEaCO_2_ and eTEaCO_2_ plots. A central control unit regulated the CO_2_ concentration in the eCO_2_ plots using a GMT222 sensor (Vaisala, Helsinki, Finland) positioned at the center of each plot. This sensor measured the concentration to maintain a set level of 600 ppm by employing a PID algorithm that utilized the CO_2_ difference between the aCO_2_ and eCO_2_ plots, along with wind speed data.

Throughout the experiment, plants were irrigated with sprinklers to ensure soil moisture levels remained near field capacity. Soil moisture and soil temperature were monitored using, respectively, ML2X and ST2 sensors, which were installed in the center of each plot at a depth of 10 cm and connected to a DL2 datalogger (Delta-T Devices Ltd., Burwell, Cambridge, UK).

During the experiment conducted from January to June 2015, the average daytime and nighttime temperatures recorded were as follows: 23.8 °C/14.9 °C for the Control treatment, 24.3 °C/15.0 °C for the elevated CO_2_ (eCO_2_) treatment, 25.3 °C/16.7 °C for the elevated temperature (eTE) treatment, and 25.8 °C/16.7 °C for the combined elevated temperature and elevated CO_2_ (eTEeCO_2_) treatment. The slight increase in temperature for the eCO_2_ treatment compared to the Control may be associated with stomatal closure caused by the elevated CO_2_ levels, as noted by (Habermann et al., 2019 [29]). The average ambient CO_2_ concentration in the control plots was 395 ± 15 ppm, while the Free Air Carbon Dioxide Enrichment (FACE) fumigation increased the daytime average CO_2_ concentration to 595 ± 20 ppm in the eCO_2_ and eTEeCO_2_ plots during the course of the experiment. Although CO_2_ fumigation was not performed at night, the average nighttime CO_2_ concentration was 455 ± 19 ppm, primarily due to heterotrophic respiration from plants and soil. Over the duration of the study, the average soil moisture in the Control and eTE treatments was recorded at 0.30 ± 0.07 m^3^ m^−3^. Under the eCO_2_ treatment, soil moisture increased to 0.34 ± 0.06 m^3^ m^−3^, while in the combined eTEeCO_2_ treatment, it averaged 0.29 ± 0.07 m^3^ m^−3^. Soil temperatures in the warmed treatments (eTEaCO_2_ and eTEeCO_2_) were approximately 1 °C higher than those in the unwarmed treatments (aTEaCO_2_ and aTEeCO_2_) [29]. A detailed description of this system can be found in (Martinez et al., 2014 [30]) and (Prado et al., 2016 [31]), as well as in [29].

- Flowers and seeds sampling

At the beginning of the flowering period of the experiment (Figure 1b,c), 21 maternal plants of *S. capitata* from each treatment were labeled and leaf samples were collected. However, mature inflorescences (Figure 1d) were collected from a smaller subset of these individuals, as detailed below, and stored in paper bags.

#### 2.2.2. Laboratory Analyses

The experimental field setup followed a randomized block design with four replicates per treatment. Inflorescences were sampled from these field replicates, but for laboratory analyses, pods and seeds were subsequently pooled by treatment group.

Seeds of *S. capitata* were manually extracted from their pods (see Figure 1e–g) for the quality assessments outlined below. To examine the impact of storage duration on germination performance, the inflorescences were transferred to hermetically sealed plastic bags at room temperature (20–25 °C) for periods of 32, 40, and 71 weeks.

- Seed production and quality

For pod seed abortion (% empty pods), non-viable seeds (%), and hundred-seed weight (HSW), data were collected from 16 maternal plants per treatment. Each plant contributed 100 pods, from which mean values for pod abortion and seed viability were individually calculated. Seed selection and quality assessments were performed visually under stereoscopic magnification (Leica S4E, 2.5×; Leica Microsystems GmbH, Wetzlar, Germany). From each plant, 100 healthy seeds—defined by intact seed coats, absence of physical damage or deformation, and uniform morphology—were manually selected and weighed using a precision scale (0.01 mg) to determine HSW. Additionally, mean seed number per inflorescence (SPI) was calculated from 10 maternal plants per treatment, each contributing 10 inflorescences. For each plant, SPI was obtained as the average number of seeds across its 10 sampled inflorescences. These plant-level means were used as independent observations.

- Seed Coat Color Classification

Before each germination analysis conducted at 32, 40, and 71 weeks, seeds were preserved within the inflorescences. This conservation method may have contributed to maintaining their coat color until the final evaluation. Seeds were then manually extracted from the inflorescences and pods for posterior analysis. For each storage period × treatment combination, seeds from four maternal plants were examined, totaling 12 maternal plants per treatment. From each maternal plant, 100 visually healthy seeds were randomly selected and initially assessed for seed coat coloration. Classification was performed under stereoscopic magnification (Leica S4E, 1.25×) due to the small seed size (~2 mm; see Figure 1f). The observed colors were consistently classified across all experiments. At the conclusion of all germination assessments, seeds with similar coat colors within each treatment were grouped, counted, and expressed as percentages.

- Seed Germination Analysis

Inflorescences were stored at room temperature (20–25 °C) for 32, 40, and 71 weeks to simulate both medium- and long-term storage scenarios under ambient conditions and assess their effects on germination performance. The same seeds previously classified by coat color were subsequently re-mixed and used for germination trials, ensuring biological consistency while allowing an unbiased assessment of seed viability. Thus, for each storage period × treatment combination, seeds from four maternal plants were used, totaling 12 maternal plants per treatment. In each trial, 100 visually healthy seeds were selected per maternal plant, resulting in a total of 4800 seeds across all treatments (100 seeds × 4 maternal plants × 3 storage periods × 4 treatments).

*S. capitata* seeds exhibit physical dormancy due to the impermeability of the seed coat, resulting in low germination rates and a high proportion of hard seeds (93% to 100%) (Battistin, 1984 [32]). This makes dormancy-breaking treatments essential. Accordingly, prior to each in vitro germination experiment, light mechanical scarification was performed using fine sandpaper (particle diameter: 68 µm; grit size: p220) to facilitate water uptake and overcome the physical barrier to germination, following the procedures of (McIvor 1976, [33]), (Rodrigues et al., 2010 [34]), and (Chaves et al., 2017 [35]). However, scarified seeds may still retain physiological dormancy, which gradually diminishes during post-harvest aging [35]. Scarification served to standardize germination conditions, ensuring that any observed differences among treatments were attributable to experimental factors rather than variation in physical dormancy.

In each test, 100 seeds per maternal plant were placed on sterile filter paper within Petri dishes (Brasil-MAPA, 2009 [36]). The paper was kept moist with sterile distilled water throughout the test. The experimental conditions included a 12 h photoperiod, an average temperature of 25 °C [35], and a relative humidity of 50%. Germination evaluation was conducted daily for eight days, during which more than 90% of the seeds had germinated in most treatments [36]. The parameters assessed included the germination speed index (GSI), calculated using the formula: GSI = (G1/D1) + (G2/D2) + … (Gn/Dn), where G1, G2 … G100 represent the number of seeds germinated in the first, second, and total count on each day, respectively. D1, D2 … Dn is the number of days from the first, second, until the count after germination (Maguire 1962 [37]). Additionally, the average germination time (GT) [t = Σ*ni.ti*/Σ*ni*, where *ti* is the time from the start of the experiment to the *i*th observation (day), and *ni* is the number of seeds germinated in the time *i* (not the accumulated number, but the number correspondent to the *i*th observation)] according to (Labouriau and Valadares 1976 [38]). Lastly, the germination percentage (Gr, %), determined by the number of seeds germinated on the last day of the experiment divided by the total number of seeds multiplied by 100 (Ferreira and Borghetti, 2004 [39]).

- Abnormal germinated seedlings

Germinated seedlings in each experimental group were visually assessed and categorized into two groups: high-vigor seedlings and low-vigor seedlings. High-vigor seedlings displayed healthy, well-developed stems and cotyledons and were notably larger in size. These seedlings were the same ones analyzed for germination percentage (Gr). Low-vigor seedlings exhibited traits such as twisted stems, yellowish cotyledons, and reduced size compared to healthy seedlings. Evaluations were based on the same 12 maternal plants per treatment used in the germination and seed coat color analyses, encompassing four plants per storage period × treatment. In each test, 100 visually healthy seeds per maternal plant were germinated and monitored for seedling vigor. Due to the low number of abnormal seedlings observed at each time point (32, 40, and 71 weeks of storage), abnormal seedlings were evaluated collectively and expressed as percentages.

- Genetic diversity analysis between generations

Deoxyribonucleic Acid (DNA) Extraction, Amplification, and Electrophoresis. We analyzed genetic diversity across generations using 10 maternal plants and their progeny for each treatment. Leaves were collected from *S. capitata* maternal plants in the field, and we subsequently germinated 16 seedlings in the laboratory, resulting in a total of 160 progeny individuals per treatment. The samples were stored at −20 °C until DNA extraction, which was conducted using an adapted Cetyltrimethylammonium bromide (CTAB) protocol for genomic DNA extraction, as described by (Alzate-Marin et al., 2009 [40]). The DNA was quantified using a NanoDrop Spectrophotometer (Thermo Scientific, Waltham, MA, USA).

Simple Sequence Repeats (SSRs), also known as microsatellites, were used as molecular markers due to their high polymorphism and codominant inheritance [41]. Based on these properties, genomic DNA samples were amplified using seven SSR primers selected from a previously validated set of fifteen [42,43] (see File S1—Table S1). Primer selection was based on amplification quality, polymorphism, and compatible annealing temperatures, which enabled duplex polymerase chain reaction (PCR) according to fragment sizes, following protocols validated by (Alzate-Marin et al., 2019 [44]) (File S1—Table S1). Duplex reactions were performed for the primer pairs: SC18-01 A2A/E4, SC18-01T G9/F2, SC18-01T G12A/SC18-01 H5, and SC18-02 E12. PCRs were conducted in 12 µL volumes using the GoTaq^®^ Kit (Promega, Madison, WI, USA), consisting of 5 µL nuclease-free water, 5 µL master mix (including 400 nM of each deoxyribonucleotide triphosphate —dNTPs— and 3.0 mM MgCl_2_), 1 µL of each primer, and 2.0 ng/µL of genomic DNA.

Amplifications were performed in a Mastercycler^®^ pro S thermocycler (Eppendorf, Hamburg, Germany) under the following conditions: initial denaturation at 94 °C for 5 min; 30 cycles of denaturation at 94 °C for 60 s, annealing at 60 °C for 60 s, and extension at 72 °C for 60 s; followed by final extension at 72 °C for 7 min. PCR products were analyzed using the GelBot^®^ automated capillary electrophoresis system (BiOptic Inc., New Taipei City, Taiwan; Loccus, Cotia, SP, Brazil), equipped with a high-resolution cartridge for fragment separation (2–1000 bp). Data analysis was conducted using Q-Analyzer software (BiOptic Inc., New Taipei City, Taiwan).

Genotypic data were analyzed locus by locus from a total of 10 maternal plants per treatment, each contributing 16 progeny individuals (160 offspring per treatment). Each of the seven SSR markers served as an independent replicate for molecular comparisons across treatments. These multilocus profiles formed the basis for diversity and population structure analyses.

- Statistical analyses

Data normality was evaluated using the Shapiro–Wilk test. Percentage data were transformed using the arcsine square root method. For other non-normally distributed data, a Box–Cox transformation with a lambda (λ) value of 0.05 was applied to enhance the data distribution. A two-way Analysis of Variance (ANOVA) (*p* < 0.05) was used to evaluate the effects of temperature and CO_2_—each tested at two levels (ambient and elevated)—as well as their interaction. This analysis was applied to physiological and genetic traits, including seeds per inflorescence (SPI), hundred-seed weight (HSW), percentage of seed abortion, percentage of non-viable seeds, percentage of abnormal seedlings, and genetic diversity metrics, using data from all four treatment combinations (aTEaCO_2_, aTEeCO_2_, eTEaCO_2_, eTEeCO_2_). Additionally, two separate three-way ANOVAs (*p* < 0.05) were performed, both including the same treatment combinations: one to assess germination parameters (GSI, GT, Gr%), incorporating storage duration (week) as the third factor, and another to evaluate seed coat color (%), using color category as the third factor. Effect size (η^2^) for each factor was calculated as the ratio of SS_effect to SS_total (η^2^ = SS_effect/SS_total). When significant main effects or interactions between factors were identified, treatment means were compared using post hoc Tukey or Šídák tests at a 5% significance level. Statistical analyses were carried out using the software PAST version 4.03 [45] and GraphPad Prism version 8.0.1 (GraphPad Software, San Diego, CA, USA).

We calculated genetic diversity parameters across generations and treatments, including the average (A) and effective (Ae) number of alleles per locus, observed (Ho) and expected (He) heterozygosity, and inbreeding coefficient (*Fis*). These calculations were performed using GenALEx software version 6.51b2 [46]. We compared the mean values of the maternal group to those of the progeny group for each parameter within each treatment. Since some parameters did not meet normality assumptions, we adopted a fully non-parametric approach using the Mann–Whitney test with the software PAST to ensure analytical consistency [45]. Additionally, we employed GenALEx to assess genetic structure by quantifying the distribution of genetic diversity between maternal and progeny populations through analysis of molecular variance (AMOVA), principal coordinate analysis (PCoA), and the genetic differentiation index (*Fst*). Statistical significance for AMOVA analyses was determined through 999 bootstrap replicates.

## 3. Results

### 3.1. Production and Seed Quality

Elevated temperature consistently influenced key reproductive traits (Table 1 and File S2—Tables S1–S4). It significantly increased seed production per inflorescence -SPI (F(1,36) = 12.80; *p* = 0.0010; η^2^ = 0.256), raising values from 14.12 ± 1.24 (control) to 20.62 ± 1.18—an increase of approximately 46.0%. Seed abortion rates also declined under elevated temperature (F(1,60) = 6.52; *p* = 0.013; η^2^ = 0.0964), decreasing from 9.75 ± 1.09% to 5.56 ± 0.98% (−42.9%). Likewise, proportion of non-viable seeds was significantly reduced (F(1,60) = 8.69; *p* = 0.004; η^2^ = 0.123), falling from 15.62 ± 2.65% (control) to 6.69 ± 1.10% (−57.2%).

In contrast, none of the tested factors—temperature, CO_2_ concentration, nor their interaction—significantly influenced the mass of 100 seeds, indicating this parameter remains stable across treatments. These findings underscore that while temperature plays a decisive role in enhancing reproductive efficiency and seed quality, it does not affect seed biomass, suggesting that reproductive gains occur without compromising seed development (see Table 1 and File S2—Tables S1–S4).

### 3.2. Coat Seed Colors

**Figure 2:**
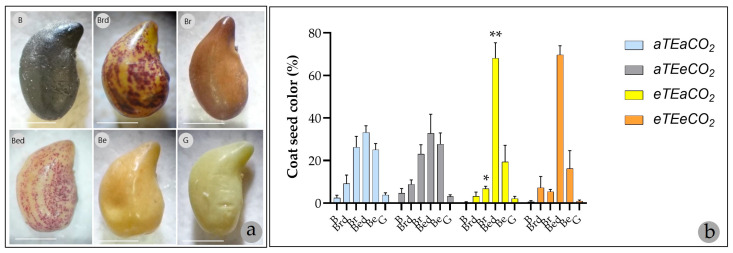
(**a**) The color classes of the integument of *S. capitata* seeds are classified as: B = black; Brd = brown with dots; Br = brown; Bed = beige with dots; Be = beige; G = green. Scale bar: 1 mm (1.25×, Leica S4E Microscope, Leica Microsystems GmbH, Wetzlar, Germany). (**b**) Mean values of seeds in each coat color class in each maternal treatment in comparison with control (aTEaCO_2_) (* *p* < 0.05; ** *p* < 0.01, Šídák multiple comparisons test).

The seed coat colors observed in *S. capitata* integument included black (B), brown with dots (Brd), brown (Br), beige with dots (Bed), beige (Be), and green (G) as illustrated in Figure 2a. Although temperature had a modest overall effect (F(1,48) = 4.177; *p* = 0.0465; η^2^ = 0.0097), its interaction with seed coat phenotype was highly significant (F(5,48) = 11.90; *p* < 0.0001; η^2^ = 0.1382) (Table 2 and File S2—Table S5), suggesting that thermal conditions selectively enhance the expression of specific seed colors already intrinsic to the species.

Elevated temperature significantly altered the distribution of seed coat types, increasing the percentage of beige with dots (Bed) seeds by 104.98% and decreasing brown (Br) seeds by 20% compared to the control (Figure 2b). A similar pattern was observed under combined elevated temperature and CO_2_ conditions, resulting in a 108.94% increase in Bed seeds (Table 2, Figure 2b). As no significant interaction between elevated temperature and elevated CO_2_ was detected (all *p* > 0.83; η^2^ < 0.002), these changes are attributed solely to warming (Table 2 and File S2—Table S5).

### 3.3. Seed Germination

The germination speed index (GSI) measures how quickly seeds germinate over time, with higher values indicating faster germination. Germination Time (GT) represents the average time it takes for seeds to germinate, making it a useful parameter for comparing different treatments or environmental conditions. The Germination percentage (Gr, %) evaluates the percentage of seeds that successfully germinate, directly reflecting seed viability. Together, these indices provide a comprehensive assessment of both the efficiency and overall effectiveness of germination processes.

#### 3.3.1. Effect of Warming and Elevated CO_2_ on Germination

Elevated temperature significantly enhanced germination dynamics across all measured traits (Table 3 and File S2—Tables S6–S8). Seeds exposed to warming exhibited a 23.5% higher germination speed index (GSI = 66.8 ± 5.61 vs. 54.07 ± 6.17 from control) and an 11.2% greater germination percentage (Gr% = 95.08 ± 1.37 vs. 85.50 ± 3.50 from control), supported by statistically significant effects (GSI: F(1,36) = 10.90, *p* = 0.0022, η^2^ = 0.048; Gr%: F(1,36) = 11.85, *p* = 0.0015, η^2^ = 0.143). Germination timing (GT) was also accelerated, with average time reduced by 9.6% (from 2.72 ± 0.25 in control to 2.46 ± 0.25 days) under combined warming and elevated CO_2_ (GT: F(1,36) = 6.75, *p* = 0.014, η^2^ = 0.028). This interaction suggests optimal environmental conditions that may improve water uptake and energy availability for the emerging seedling. These findings indicate that thermal stress acts as a physiological trigger, promoting faster and more successful seed emergence.

In contrast, elevated CO_2_ alone did not significantly affect any germination parameter (all *p* > 0.10; η^2^ < 0.07), indicating that atmospheric enrichment had little influence on early seed performance under the tested conditions (Table 3 and File S2—Tables S6–S8).

#### 3.3.2. Effects from Both Storage Time and Treatments

**Figure 3:**
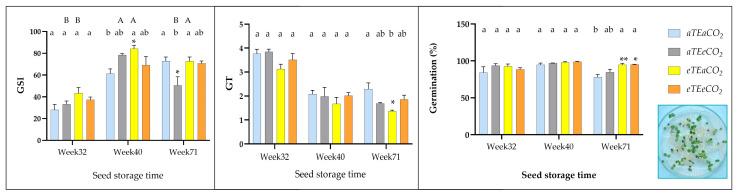
GSI—germination speed index, GT—germination time, Germination percentage (Gr, %) of *S. capitata* seeds from plants derived at different levels of temperature and CO_2_ concentration. Columns show average values, with bars indicating the standard error of the mean (* *p* < 0.05; ** *p* < 0.01, Tukey multiple comparisons test).

Seed germination was strongly influenced by temperature and storage duration across all measured traits. Significant effects were observed for both treatment and storage time (weeks), as reflected in all germination parameters (Table 3 and File S2—Tables S6–S8, Figure 3).

Storage duration was the most impactful factor, with highly significant week-based effects for germination speed index (GSI: F(2,36) = 75.60; *p* < 0.0001; η^2^ = 0.676), Germination Time (GT: F(2,36) = 92.20; *p* < 0.0001; η^2^ = 0.765), and Germination Percentage (Gr%: F(2,36) = 10.92; *p* = 0.0002; η^2^ = 0.263), indicating progressive shifts in germination dynamics over time—potentially driven by physiological readiness or seed maturation processes (Table 3 and File S2—Tables S6–S8). Notable interaction effects emerged for GSI, where a three-way interaction among elevated temperature, elevated CO_2_ and storage time was significant (F(2,36) = 8.05; *p* = 0.0013; η^2^ = 0.072), and for GT, where an elevated temperature and elevated CO_2_ interaction was detected (F(1,36) = 6.75; *p* = 0.0135; η^2^ = 0.028), suggesting environment-dependent modulation of germination behavior (Table 3 and File S2—Tables S6–S8).

For GSI, the lowest mean value occurred at 32 weeks of storage (35.5 ± 3.24), while significantly higher values were observed at 40 weeks (73.27 ± 5.10) and 71 weeks (66.72 ± 5.40) (Figure 3, File S1—Table S2). Under the warming treatment, the highest mean GSI was recorded at 40 weeks (84.20 ± 3.14), compared to the control (61.32 ± 4.40). Conversely, at 71 weeks, the elevated CO_2_ treatment showed reduced values (50.56 ± 8.00) relative to the control (72.81 ± 3.80) (Figure 3). Regarding GT, the mean germination time was highest at 32 weeks (3.56 ± 0.16), declining significantly by 40 weeks (1.94 ± 0.09) and continuing to decrease at 71 weeks (1.80 ± 0.19) (Figure 3, File S1—Table S2). The lowest GT value under warming was observed at 71 weeks (1.37 ± 0.05), compared to the control (2.30 ± 0.26) (Figure 3). For Gr%, no significant differences were found across storage times (mean = 91.56 ± 1.45%) (Table 3). However, at 71 weeks, both the warming treatment (94.75 ± 2.0%) and its combination with elevated CO_2_ (95.0 ± 0.0%) yielded higher germination rates than the control (77.75 ± 4.0%) (File S1—Table S2, Figure 3).

#### 3.3.3. Abnormal Seedlings

The occurrence of abnormal seedlings was significantly reduced in response to elevated temperature, as indicated by a two-way ANOVA (F(1,44) = 8.818; *p* = 0.0048, η^2^ = 0.1653), confirming temperature as a major factor contributing to improved seedling quality (Table 3 and File S2—Table S9). The lowest abnormality rate was recorded in the group associated with elevated temperature under ambient CO_2_ conditions (1.33 ± 0.48%), compared to higher rates observed in the control group (2.92 ± 0.63%). This corresponds to a 54.5% reduction in abnormal seedling occurrence, suggesting that elevated temperatures during developmental stages positively influence seedling vigor. These findings are consistent with the germination percentage data, which reflects only vigorous seedlings, implying that heat-associated conditions may enhance overall seed quality and reduce the expression of developmental defects. Neither CO_2_ concentration nor its interaction with temperature contributed significantly to the variation in abnormal seedlings (*p* > 0.48; η^2^ < 0.009), reinforcing the conclusion that temperature alone shaped this outcome (Table 3 and File S2—Table S9).

### 3.4. Effects of Warming, Elevated CO_2_, and Their Interaction on Genetic Diversity Parameters in S. capitata

#### 3.4.1. Frequency Analysis

Mother plants had a total of 54 alleles: 43 in the control group, 37 in the eCO_2_ group, 32 in the eTE group, and 39 in the eTEeCO_2_ group. In comparison, the progenies exhibited a total of 59 alleles, with 46 in the control group, 45 in the eCO_2_ group, 39 in the eTE group, and 44 in the eTEeCO_2_ group. This suggests that the eCO_2_ treatment preserved more alleles than the eTE treatments (see File S1—Table S3). The most stable alleles between maternal plants and their progenies, indicating genetic resilience, included A2A-238, 240, and 242; E4-304 and 308; F2-194 and 200; G9-240 and 248; G12-266 and 270; E12-292, 298, 318, 322, and 328; and H5-200 and 202. These alleles remained largely unchanged, highlighting their essential role in maintaining genetic stability within the population. However, allele E12-292 showed a decrease, while E12-322 increased under the eTE treatment.

In the eCO_2_ treatments, the alleles F2-192 and G12A-254 in progeny exhibited a decline compared to the maternal plants. In the eTE treatments, some alleles increased in frequency, such as E12-292 and E12-322, while others decreased, including E4-318, F2-204, E12-288, E12-296, and H5-208. In the combined treatments (eTEeCO_2_), certain alleles showed an increase in frequency, such as G12A-266 and E12-322, while others declined, including A2A-228, A2A-242, E4-298, E4-318, F2-194, F2-204, F2-208, G9-258, G12A-272, E12-337, and H5-204. Notably, E12-322 increased in both warming treatments, while E4-318 and F2-204 decreased in both conditions.

The findings suggest that selective pressure, particularly in the eTE and combined treatments, favors certain genetic variants while also causing genetic losses, which could reduce overall diversity. In the control group, some alleles increased, such as G12A-272 and E4-304, while others declined, including H5-208, G12A-278, G9-258, E4-318, E4-326, and F2-190. This indicates natural genetic fluctuations can occur even without environmental stressors. Gene flow was observed across all treatments, with alleles that were not originally present in some maternal groups appearing in the progeny from others. Notable examples include A2A-234 and H5-204 in the eCO_2_ treatment, F2-192 and F2-208 in eTE, E4-296 and F2-196 in combined treatments, and F2-204 and G12A-260 in the control group. Additionally, the alleles F2-197 (eTE), E4-311 and E12-330 (eCO_2_), E4-316 (eTEeCO_2_), and G9-246 (Control) were found only in the progeny, although at low frequencies. This may be due to gene flow from non-sampled maternal plants (see File S1—Table S3).

#### 3.4.2. Genetic Parameters Analysis of Maternal Plants vs. Progeny

The non-parametric analysis indicates that there were no statistically significant differences in the maternal and progeny means for the genetic parameters (A, Ae, Ho, He, and *Fis*) within each treatment group (Control, eCO_2_, eTE, and their combination), as demonstrated by the Mann–Whitney test. This finding suggests genetic stability across generations (see Table 4 and File S1—Table S4). Further analyses conducted within each generation reveal that observed heterozygosity (Ho) was lower than expected heterozygosity (He) (refer to Table 4 and File S1—Table S5). This implies that these groups have a higher proportion of homozygotes than one would anticipate in a population at Hardy–Weinberg equilibrium (File S1—Table S5).

In the eTE treatment, Ho decreased in progeny (from 0.40 in maternal plants to 0.28 in progeny), indicating reduced genetic diversity and increased homozygosity (Table 4). Expected heterozygosity (He) remained relatively stable across all treatments, indicating that overall genetic diversity was preserved, with a mean of 0.70 in maternal plants and 0.69 in progeny. However, the He values in the eTE treatment were lower for both maternal (0.62) and progeny plants (0.61) compared to the control groups (0.77 in maternal plants and 0.76 in progeny). This suggests that the genetic variability of the initial eTE population was already reduced in the field (see Table 4).

Mean *Fis* values were positive across all experimental groups, indicating a consistent pattern of inbreeding within the populations (see Table 4). This pattern appears to be influenced by the species’ reproductive strategies. Progeny populations exhibited higher overall *Fis* values (0.58) compared to maternal plants (0.50), suggesting that inbreeding persisted or even intensified following reproduction. Notably, the control progenies showed elevated *Fis* values, which contributed to the group’s mean. A particularly pronounced difference was observed in the eTE treatment, where *Fis* increased from 0.37 in maternal plants to 0.59 in progenies, indicating a substantial rise in inbreeding levels under warming.

#### 3.4.3. ANOVA Analysis

Most genetic diversity parameters assessed in the progeny—namely average number of alleles (A), observed heterozygosity (Ho), and inbreeding coefficient (*Fis*)—did not differ significantly across temperature and CO_2_ treatments, indicating a general stability under varying environmental conditions (Table 4 and File S2—Table S10). However, elevated temperature had a statistically significant effect on two functional indicators of genetic diversity. The number of effective alleles (Ae) declined under warming (F(1,24) = 4.465; *p* = 0.045; η^2^ = 0.150), and expected heterozygosity (He) was also reduced (F(1,24) = 6.296; *p* = 0.019; η^2^ = 0.191) (Table 4 and File S2—Table S10). Compared to control values (Ae = 4.60, He = 0.76), the eTE treatment showed reductions to Ae = 2.99 (−35.0%) and He = 0.61 (−19.7%), suggesting that thermal stress may diminish the genetic variability underlying adaptive potential in progeny.

These moderate effect sizes reflect a biologically meaningful shift in genotypic composition, reinforcing that elevated temperature can shape progeny-level genetic diversity even in the absence of strong directional selection. In contrast, CO_2_ concentration and its interaction with temperature had no significant impact on any parameter (*p* > 0.24; η^2^ < 0.05) (Table 4 and File S2—Table S10), indicating that elevated CO_2_ within the tested range did not affect genetic diversity outcomes.

While statistical significance was observed, it is important to note that both parameters displayed low values, similar to those in the maternal population (Table 4). Thus, this significance should be interpreted in the context of the field treatments applied.

#### 3.4.4. Genetic Structure Analyses

- PCoA, AMOVA, and *Fst* analysis among populations.

Consistent genetic structure patterns were observed across maternal and progeny samples. In mother plants, the first three PCoA axes explained 14.50%, 9.99%, and 9.44% of the total genetic variance, respectively (33.92% cumulative). Among progeny, the corresponding axes accounted for 13.97%, 11.05%, and 10.43%, totaling 35.46% of explained variance. Despite differences in sample size, both generations exhibited comparable resolution within the ordination space.

**Figure 4:**
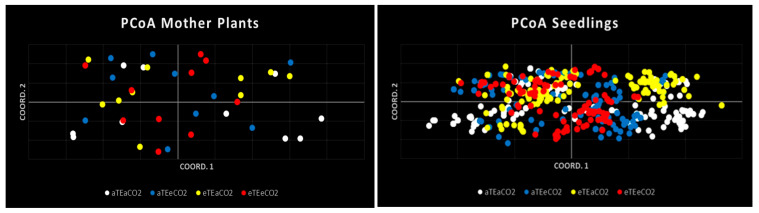
Principal Coordinates Analysis (PCoA) of two generations of *S. capitata*.

This resolution supports the interpretation of subtle differences in genetic structuring among environmental treatments. The PCoA plot (Figure 4) showed that all four populations occupied similar regions in the ordination space; however, variations in individual distribution patterns were evident. Non-warming treatments (aTEaCO_2_ and aTEeCO_2_) displayed more continuous dispersion, suggesting lower genetic structuring and broader retention of genetic variability. In contrast, the elevated temperature plus elevated CO_2_ group (eTEeCO_2_) showed slight clustering, indicating an emerging pattern of genetic structuring. This was more pronounced under elevated temperature with ambient CO_2_ (eTEaCO_2_), where two distinct genetic clusters were observed. These findings point to increased genetic structuring under warming treatments, potentially driven by thermal selection. Nonetheless, all populations remained partially overlapping in PCoA space, suggesting that while selection pressures may shape genetic composition, they do not lead to complete differentiation among treatment groups.

The AMOVA (Analysis of Molecular Variance) results for maternal populations show that 99% of genetic variation occurs within populations, while only 1% can be attributed to differences among them (see Table 5). The estimated variance of 0.061, and the non-significant *p*-value of 0.304 indicates minimal genetic differentiation. This uniformity likely reflects their shared origin from the same seed lot.

Conversely, progeny populations exhibited greater differentiation: 91% of variation occurred within populations, while 9% was significantly attributed to differences among them (Est. Var = 0.784; *p* = 0.001). These findings indicate the emergence of genetic structuring, likely driven by environmental pressures, selection, or genetic drift during treatment. When maternal and progeny populations were analyzed together, AMOVA revealed that 8% of the variance was significantly due to population differences, while 92% remained within populations (Est. Var = 0.704; *p* = 0.001). These results support the trend observed in the PCoA: increased genetic structuring in progeny compared to mothers, though genetic diversity remains primarily within populations.

Building on the findings from the AMOVA analysis, the *Fst* analysis offers further insights into genetic structure across different generations (Table 6).

The mean *Fst* value of 0.06 indicates moderate genetic differentiation, suggesting that there is selection pressure while still allowing for gene flow, particularly in the eTEaCO_2_ treatments (File S1—Table S6). Additionally, the Nm estimate sheds light on genetic connectivity between maternal and progeny populations. The maternal Nm value of 3.713 reflects the initial genetic connectivity established from the commercial seed lot, as all maternal plants originated from a common genetic pool before being placed in the experimental environment. Once planted in the field, pollination facilitated genetic exchange, influencing the diversity seen in their progeny. Conversely, the progeny Nm value of 3.843 measures the gene flow occurring among maternal plants from different treatments through pollination. The combination of a moderate *Fst* and a high Nm indicates that genetic connectivity remains strong, allowing progeny to maintain shared genetic diversity while also experiencing a degree of differentiation.

## 4. Discussion

Understanding the effects of temperature and elevated CO_2_ (eCO_2_) on native and agricultural species is critical for conservation and food security. Yet, studies on tropical species remain scarce. In parallel field experiments, we exposed maternal plants of *Stylosanthes capitata* to warming (+2 °C) and elevated CO_2_ (600 ppm) under irrigated conditions. Warming increased flower number and accelerated flowering onset, enhancing attractiveness to pollinators. Despite these shifts, outcrossing rates remained consistent across treatments [6]. Conversely, warming also triggered premature degeneration of tapetal cells in pollen sacs [23], potentially impairing seed formation—a limitation likely offset by increased floral output [6]. Building on these findings, our study evaluates, under laboratory conditions, how warming (+2 °C) and elevated CO_2_, experienced by maternal plants, affect progeny physiology, genetic diversity, and population structure in *S. capitata*.

We hypothesized that warming would increase seed production per inflorescence, based on its stimulatory effects on flowering and pollinator visitation [6,24]. This was confirmed, likely due to enhanced floral output [6], which sustained seed yield even under reproductive stress. Regarding reproductive anomalies, we expected similar abortion rates across treatments, given that abnormalities were previously observed even under control conditions [23]. However, warming reduced abortion and non-viable seed formation, suggesting developmental failures were compensated by increased floral volume—rejecting the hypothesis of uniform abortion rates and highlighting an indirect benefit of warming.

We also hypothesized that warming and elevated CO_2_ would affect seed traits—specifically seed mass, coat color, and germination dynamics. This was partially confirmed. Warming modified coat color and reduced abnormalities, both linked to improved viability, though seed mass remained unchanged. Germination was enhanced by warming, maintained performance over time, with modest gains from warming × eCO_2_ interaction. In contrast, long-term CO_2_ exposure impaired germination (GSI), indicating reduced physiological resilience—thus partially confirming the hypothesis and positioning warming as the key driver of seed performance.

Finally, we hypothesized that warming would increase progeny genetic diversity by stimulating flowering and promoting outcrossing via pollinator visitation [6,24]. This was only partially supported. Although seed production increased (SPI), diversity metrics—such as expected heterozygosity (He) and effective allele number (Ae)—did not improve and were negatively affected. These findings suggest increased geitonogamy [6] and enhanced genetic structuring under warming. Nevertheless, specific allelic shifts indicate possible adaptive selection in response to maternal stress, favoring traits better suited to elevated temperature and CO_2_ conditions.

Taken together, our results reveal significant alterations in reproductive, physiological, and genetic traits of *S. capitata*, confirming several key hypotheses and advancing our understanding of how future climatic conditions may shape the reproductive success and adaptability of tropical legumes.

### 4.1. Seed Quality and Germination Dynamics

Temperature is a crucial environmental factor that influences plant physiological functions, ecosystem dynamics, and biodiversity [47,48,49]. Plants thrive within specific temperature ranges, and deviations from these ranges—whether lower or higher—can affect their overall performance [50]. During reproduction, plants invest significant energy in the production of gametes and the maturation of seeds [51]. Seed development relies on photosynthetic activity in the leaves, which provides essential nutrients for growth and resource accumulation [52,53]. Elevated atmospheric CO_2_ levels can increase seed mass by increasing the availability of assimilates. However, the effects of CO_2_ on seed mass can vary across different species and environmental conditions, including water availability [14,16,54,55,56,57]. Similarly, increases in temperature may not have any impact on seed mass or may even enhance it [58]. It is important to note that a reduction in seed mass does not necessarily indicate lower seed quality, as studies have found no consistent relationship between seed mass, germination rates, or seed vigor [11,59,60].

Our research indicates that maternal plants were affected by elevated temperatures (eTE) during their growth and reproductive phases. This means that the seeds analyzed in this study represent the cumulative effects of exposure to warming. Our findings suggest that a temperature increase of +2 °C impacts the maternal environment, influencing seed production in *S. capitata*. This influence is observed through a reduction in the number of aborted, non-viable, and abnormal seedlings, changes in seed coat coloration, and an increase in the number of seeds produced per inflorescence. Although the combination of elevated temperature and CO_2_ did not produce statistically significant results, warming still played a role in shaping the observed patterns in seed production.

Previous studies conducted by our group have demonstrated that a warming of +2 °C under irrigated conditions enhances the efficiency of photosystem II (PSII), resulting in increased leaf area and dry biomass in *S. capitata* [30]. This improvement in photosynthetic capacity likely facilitates greater resource allocation from leaves to seeds, contributing to higher seed production and viability. Similarly, (Haberman et al., 2019, [29]) performed an independent physiological study as part of the same experiment, which reported even more pronounced effects on PSII performance and antioxidant defenses. The isolated warming conditions boosted photosynthesis and starch export, while enhancements in heat dissipation and photosynthetic efficiency—suggested by the presence of carotenoids and plastoglobuli—may have supported seed maturation and quality [29]. Plastoglobuli are lipoprotein structures associated with thylakoid membranes in chloroplasts, and their occurrence often increases under abiotic stress [61]. These structures contain various compounds involved in the xanthophyll cycle, such as zeaxanthin, antheraxanthin, and violaxanthin, many of which act as reactive oxygen species (ROS) scavengers, contributing to heat dissipation [62]. Therefore, plastoglobuli accumulation likely plays a key role in plant acclimation to elevated temperatures, sustaining photosynthetic efficiency and resource flow during reproductive development, and ultimately enhancing seed viability and quality.

Warming has been found to increase the number of seeds produced. Although the interaction with elevated CO_2_ was not statistically significant, warming still showed a high effect. However, it did not enhance seed mass, whether considered alone or in combination with eCO_2_. This trend was also observed in the C_3_ grass *Austrodanthonia caespitosa* [63], suggesting a possible species-wide response to these environmental factors. Interestingly, (Bonifácio-Anacleto et al., 2024 [25]), studying *S. capitata* under warming and drought conditions in 2018, revealed that warming increased the weight of 100 seeds. This suggests that seed weight may be influenced by the conditions during the year of seed development, emphasizing the importance of collecting and preserving climatic data from the years when seeds are harvested for both agricultural and broader ecological purposes.

Elevated levels of CO_2_ alone did not result in an increase in seed number or mass, which highlights that CO_2_ enrichment does not automatically enhance reproductive traits. The positive impacts of atmospheric CO_2_ on photosynthetic productivity depend on factors such as temperature and plant genotype [64], as demonstrated in studies on rice [65]. Additionally, higher canopy temperatures and increased CO_2_ levels have been linked to pollen abnormalities [6], similar to findings in soybean [66]. These abnormalities may lead to sterility [67], potentially negating the expected improvements in yield. While CO_2_ enrichment enhances photosynthetic efficiency in *S. capitata* (as reported by [29]), the determination of reproductive traits like seed size and mass is influenced by a complex interaction of physiological and environmental factors, which often outweigh the benefits driven by CO_2_ [68].

### 4.2. Germination

Fast and successful germination is crucial for the survival of species and helps improve seedling establishment under field conditions [69]. Maternal temperature significantly influences seedling emergence, with long-term effects that may become more pronounced due to global warming [70,71]. The process of seed germination is regulated by a delicate hormonal balance, primarily between abscisic acid (ABA), which helps maintain dormancy, and gibberellin (GA), which promotes germination and growth. The protein ABA Insensitive 5 (ABI5), a basic leucine zipper transcription factor, plays a central role in this regulatory process [39,72,73]. While the effects of elevated CO_2_ levels in the maternal environment on germination are still inconclusive, studies have shown highly variable responses across different plant functional groups and species [17].

Our findings indicate that the longevity of *S. capitata* seeds varied during storage, exhibiting treatment-dependent responses that were influenced by the environmental conditions present during seed development. Maternal warming helped preserve seed viability, allowing for high germination rates of approximately 95% for up to 71 weeks. Additionally, this warming accelerated germination speed, which peaked at 40 weeks (germination speed index = 84). Research on *Viscaria alpina* suggests that the effects of maternal warming on seed longevity are affected by water availability [74]. (Probert et al., 2019 [75]) further demonstrated that seeds originating from hot, dry maternal environments display greater longevity than those from cooler, wetter conditions. Notably, our seeds developed during a particular drier period, which aligns with these findings and supports the notion that environmental conditions during seed development can significantly shape long-term seed survival strategies.

Additionally, warming alone reduced germination time by 24%, and when combined with eCO_2_, it reduced germination time by 10%. These results are consistent with observations made by (Marty & Bassirirad 2014 [17]). The most significant effect was noted at 71 weeks, where germination time decreased to 1.37 days. These findings align with the results reported in [25], which indicate that warming accelerated germination in *S. capitata* during the first five days when using non-scarified seeds. This suggests that temperature conditions during seed development influence physiological processes that carry over into storage, thereby enhancing germination efficiency. For example, in wheat, exposure to heat during seed formation triggers molecular responses, such as the upregulation of heat shock proteins (HSP17.6 and HSP70), which are associated with cellular protection and seed vigor [76]. Conversely, our findings indicated that eCO_2_ alone negatively impacted the germination speed index (GSI) at 71 weeks. This suggests that high CO_2_ levels during seed development may have altered reserve composition or hormonal balance, hindering metabolic activation during later storage. Furthermore, these responses likely vary by species. Studies conducted under elevated CO_2_ conditions (475 ppm) show variable outcomes: no effect in *Cerastium glomeratum* and *Poa pratensis*, reduced performance in *Leontodon saxatilis*, and improved growth in *Anthoxanthum odoratum, Lolium perenne*, and *Trifolium repens* [77].

### 4.3. Seed Coat Color and Germination

Seed coat color can impact germination by affecting the seed’s permeability to water and oxygen [78]. In legumes such as *Retama sphaerocarpa* [79] and alfalfa (*Medicago sativa* L.) [80], the germination rates of yellowish seeds were found to be 30–50% higher than those of dark seeds. Additionally, maternal warming environments can influence the levels of anthocyanins and proanthocyanidins in seed coat cells, as noted in wheat (*Triticum aestivum* L.) [81]. This has significant implications for agriculture and food security. For example, legumes with varying seed coat pigmentation respond differently with respect to nitrogen fixation, thus contributing unevenly to sustainable agriculture across African soils [82].

Our findings indicate an indirect relationship between the seed coat color of *S. capitata* and germination rates. The seeds used in our experiments were predominantly beige with dots (referred to as “bed”), particularly in the warmed treatments, where their occurrence was higher (68% in eTEaCO_2_ and 69% in eTEeCO_2_). This was correlated with increased germination rates (see Table 2 and Table 3). Additionally, as noted by [25], warming resulted in a thinning of the seed coat cuticle in *S. capitata*, which expedited the imbibition process and led to faster seed germination and root development during the first five days. Similar trends were observed in germination tests with *Stylosanthes* species [32]. These results underscore the interaction between environmental conditions, maternal influences, and storage dynamics, offering valuable insights into seed longevity, morphology, and germination performance in response to future climate conditions.

### 4.4. Maternal Effects, Pollination Dynamics, and Genetic Parameters

Our analysis indicates that maternal plants exposed to warmer temperatures demonstrated low effective allele numbers (Ae) and genetic diversity (He), and these levels remained unchanged in their progeny, despite high pollinator activity during the flowering period [6]. In parallel studies conducted within the same experiment, outcrossing rates remained consistent with the control group. This suggests that key pollinators, such as *A. mellifera* and *P. lineata*—major pollinators of *S. capitata*—likely influenced genetic structuring by repeatedly visiting floral resources until they were depleted [83]. This behavior may have reinforced geitonogamy and potential endogamy [6], which could have countered any potential increases in genetic diversity and cross-pollination in the warmer treatments.

The observed changes in progeny diversity likely reflect the selection pressures that acted on maternal plants during their development. These pressures were maintained throughout the growth period until seed maturation, favoring individuals best adapted to the warmer conditions experienced in the field. This stress may have been further exacerbated by a 1 °C increase in soil temperature under elevated temperature (eTE) conditions. However, in the eTEeCO_2_ treatment, this rise in soil temperature may have been mitigated by stomatal closure induced by elevated CO_2_ levels [29]. This treatment resulted in reduced stomatal density, stomatal index, and stomatal conductance (gs), which lowered transpiration rates, increased leaf temperature, and helped preserve soil moisture throughout the growing season. These findings are supported by parallel studies from the same experiment [29]. As a result, these effects may have partially counteracted the overall impact of the combined treatment on water loss and soil moisture content.

A detailed analysis of allelic frequencies indicates that certain alleles are linked to adaptive mechanisms in response to treatment conditions observed in progeny. Significant increases in specific alleles—particularly in warmer treatments such as E12-292 in eTE, and G12A-266 in eTEeCO_2_—and the rise in allele E12-322 in both warming treatments suggest the occurrence of genetic adaptation or selection effects. This points to the persistence or expansion of progeny and potential genetic advantages under environmental stress. A similar pattern was observed in *Pinus taeda*, likely reflecting environmentally driven selection. This selection resulted from multiple shifts in allele frequencies involving alleles with moderate to small effect sizes, with a comparatively minor contribution from large-effect alleles in genes associated with moisture deficit, temperature, and precipitation [84]. However, while suggestive of environmental selection, these allelic trends must be interpreted with caution until further functional characterization is available.

Genetic differentiation was similarly low in both maternal and progeny populations (*Fst* = 0.06; AMOVA: 1% in maternal plants and 9% in progeny), indicating that environmental treatments influenced genetic outcomes without significantly reducing genetic diversity within populations. Our findings demonstrate that both genetic parameters and maternal environmental conditions work together to shape seed vigor. Although warming stress during seed development may lower genetic diversity, it also increases genetic differences, while beneficial alleles consistently improve performance under these conditions. As observed in *Brassica oleracea*, the interaction between allelic variation in ABA-related genes and environmental cues produces a range of seed responses—from rapid germination to deep dormancy—supporting a bet-hedging strategy [85]. This variability in responses, known as phenological plasticity—the ability to adjust phenology in response to environmental changes—represents a key evolutionary advantage for plants [86].

*S. capitata*, a native species of Brazilian flora, is well adapted to tropical regions that naturally experience high temperatures. It has already shown phenological shifts in response to simulated future climate scenarios [6,22,23,24,25]. As a result, changes in its geographical distribution are expected under projected climate conditions [22]. Given the limited research on seed performance in tropical species under experimental climate change scenarios, the results presented in this work provide valuable insights into the effects of warming and elevated atmospheric CO_2_ on seed quality, germination, and genetic diversity in this economically important tropical legume.

## 5. Conclusions

Elevated temperatures—and in some cases, their combination with elevated CO_2_—played a significant role in shaping both the genetic structure and physiological performance of *Stylosanthes capitata*. Early selection under warming conditions likely excluded poorly adapted individuals, reducing genetic diversity even before reproduction. Yet, progeny developed under warming exhibited improved germination, enhanced physiological efficiency, and resulted in better seed quality. In contrast, elevated CO_2_ alone showed limited or negative effects on seed viability and longevity. These results suggest that warming favored resilient genotypes capable of sustained performance, even with reduced variability. Notably, seeds from these genotypes maintained high viability after 71 weeks of storage, reinforcing their potential for long-term conservation and use. However, reduced diversity may constrain future adaptability, especially under limited gene flow or geitonogamous pollination. This highlights the importance of maintaining genetically diverse seed sources and understanding pollination dynamics to ensure crop resilience under climate change scenarios.

## Figures and Tables

**Table 1:** Analysis of variance for SPI (seeds per inflorescence), HSW (hundred seed weight), seed abortions (%), and non-viable seeds (%) of *Stylosanthes capitata* maternal plants exposed to four treatments: control—temperature and environmental CO_2_ [aTEaCO_2_]; environmental temperature and high CO_2_ (600 ppm) [aTEeCO_2_]; high temperature at +2 °C above ambient Tcanopy and environmental CO_2_ [eTEaCO_2_]; and interaction between high temperature (+2 °C above ambient Tcanopy) and elevated CO_2_ (600 ppm) [eTEeCO_2_]. Data are the mean ± SE. ns = non-significant effects of any factor; * *p* < 0.05, ** *p* < 0.01. Same letters indicate no significant difference (Tukey, *p* < 0.05). ^#^ = Analyzed using transformed data (Arcsine Square Root).

Source of Variation	Treatments (Average ± SE)				ANOVA		
	aTEaCO_2_	aTEeCO_2_	eTEaCO_2_	eTEeCO_2_	aTEeCO_2_	eTEaCO_2_	eTEeCO_2_
SPI (total number)	14.12 ± 1.24 a	15.05 ± 0.46 a	20.62 ± 1.18 b	19.50 ± 1.08 b	F = 0.179, *p* = 0.67 ns	F = 12.80, *p* = 0.001 **	F = 1.05, *p* = 0.31 ns
HSW (mg)	0.23 ± 0.008 a	0.24 ± 0.006 a	0.23 ± 0.004 a	0.22 ± 0.006 a	F = 0.0079, *p* = 0.93 ns	F = 1.14, *p* = 0.29 ns	F = 0.07, *p* = 0.79 ns
Seed abortions (%) ^#^	9.75 ± 1.09 a	8.06 ± 1.02 a	5.56 ± 0.98 b	7.31 ± 1.87 a	F = 0.033, *p* = 0.90 ns	F = 6.52, *p* = 0.013 *	F = 1.06, *p* = 0.31 ns
Non-viable seeds (%) ^#^	15.62 ± 2.65 a	12.25 ± 1.81 a	6.69 ± 1.10 b	8.75 ± 1.86 a	F = 0.09, *p* = 0.75 ns	F = 8.69, *p* = 0.004 **	F = 2.04, *p* = 0.16 ns

**Table 2:** Analysis of variance for coat seed colors (B = black; Brd = brown with dots; Br = brown; Bed = beige with dots; Be = beige; G = green) of *S. capitata* seeds from maternal plants exposed to four treatments: control — temperature and environmental CO_2_ [aTEaCO_2_]; environmental temperature and high CO_2_ (600 ppm) [aTEeCO_2_]; high temperature at +2 °C above ambient Tcanopy and environmental CO_2_ [eTEaCO_2_]; and interaction between high temperature (+2 °C above ambient Tcanopy) and elevated CO_2_ (600 ppm) [eTEeCO_2_]. Data are the mean ± SE. ns = non-significant effects of any factor; * *p* < 0.05, *** *p* < 0.001. # = Analyzed using transformed data (Arcsine Square Root).

Source of Variation	Mean Coat Color Seed % (Cc) ± SE						Factorial Analyses #			
							df	F	*p*-Value	
	B	Brd	Br	Bed	Be	G				
aTEaCO_2_	2.42 ± 1.31	9.08 ± 4.09	26.25 ± 5.14	33.25 ± 3.12	25.08 ± 2.89	3.83 ± 1.01		-	-	-
aTEeCO_2_	4.67 ± 2.19	8.83 ± 2.04	23.00 ± 4.42	32.75 ± 9.04	27.67 ± 5.31	3.08 ± 0.85	1	0.00	0.991	ns
eTEaCO_2_	0.42 ± 0.22	3.25 ± 1.94	6.75 ± 1.16	68.17 ± 7.17	19.33 ± 7.82	2.08 ± 1.10	1	4.18	0.0465	*
eTEeCO_2_	0.58 ± 0.58	7.25 ± 5.26	5.33 ± 1.08	69.67 ± 4.28	16.25 ± 8.38	0.92 ± 0.55	1	0.043	0.84	ns
Cc	2.02 ± 0.99	7.10 ± 1.35	15.33 ± 5.41	50.96 ± 10.37	22.08 ± 2.61	2.48 ± 0.63	5	63.36	*p* < 0.0001	***
Cc × aTEeCO_2_							5	0.230	0.95	ns
Cc × eTEaCO_2_							5	11.90	*p* < 0.0001	***
Cc × eTEeCO_2_							5	0.171	0.972	ns

**Table 3:** Analysis of variance for (a) germination speed index (GSI), (b) germination time (GT), (c) germination percentage (Gr-%) and (d) abnormal seedlings (%) of *S. capitata* exposed to four treatments: control-temperature and environmental CO_2_ [aTEaCO_2_]; environmental temperature and high CO_2_ (600 ppm) [aTEeCO_2_]; high temperature at +2 °C above ambient Tcanopy and environmental CO_2_ [eTEaCO_2_]; and interaction between high temperature (+2 °C above ambient Tcanopy) and elevated CO_2_ (600 ppm) [eTEeCO_2_]. Data are the mean ± SE. ns = non-significant effects of any factor; * *p* < 0.05, ** *p* < 0.01, *** *p* < 0.001, ns = Not significant, # = Analyzed using transformed data (Arcsine Square Root).

Source of Variation	Mean ± (SE)	Factorial Analyses			
		df	F	*p*-Value	
(a) Germination speed index (GSI)					
aTEaCO_2_	54.07 ± 6.17	-	-	-	-
aTEeCO_2_	54.10 ± 6.19	1	2.08	0.157	ns
eTEaCO_2_	66.80 ± 5.61	1	10.9	0.0022	**
eTEeCO_2_	59.03 ± 5.32	1	2.11	0.155	ns
Weeks	58.50 ± 11.64	2	75.6	0.0001	***
Week × aTEeCO_2_		2	2.43	0.103	ns
Week × eTEaCO_2_		2	0.149	0.86	ns
Week × eTEeCO_2_		2	8.05	0.0013	**
(b) Germination time (GT)					
aTEaCO_2_	2.72 ± 0.25	-	-	-	-
aTEeCO_2_	2.51 ± 0.31	1	0.69	0.41	ns
eTEaCO_2_	2.06 ± 0.25	1	8.90	0.0051	**
eTEeCO_2_	2.46 ± 0.25	1	6.75	0.014	*
Weeks	2.44 ± 0.57	2	92.9	*p* < 0.0001	***
Week × aTEeCO_2_	-	2	0.49	0.62	ns
Week × eTEaCO_2_	-	2	0.56	0.57	ns
Week × eTEeCO_2_	-	2	1.02	0.371	ns
(c) Germination percentage (Gr-%) #					
aTEaCO_2_	85.50 ± 3.50	-	-	-	-
aTEeCO_2_	91.58 ± 2.16	1	0.68	0.413	ns
eTEaCO_2_	95.08 ± 1.37	1	11.85	0.0015	**
eTEeCO_2_	94.08 ± 1.48	1	3.562	0.07	ns
Weeks	91.56 ± 1.45	2	10.92	0.0002	***
Week × aTEeCO_2_	-	2	0.19	0.981	ns
Week × eTEaCO_2_	-	2	3.288	0.048	*
Week × eTEeCO_2_	-	2	1.104	0.343	ns
(d) Abnormal Seedlings (%) #					
aTEaCO_2_	2.92 ± 0.63	-	-	-	
aTEeCO_2_	3.50 ± 0.87	1	0.49	0.48	ns
eTEaCO_2_	1.33 ± 0.48	1	8.82	0.0048	**
eTEeCO_2_	1.75 ± 0.63	1	0.025	0.87	ns

**Table 4:** Analysis of means and variance for genetic diversity parameters (A: average number of alleles, Ae: effective number of alleles, Ho: observed heterozygosity, He: expected heterozygosity, *Fis*: fixation index) in the mother plants and their seedling populations of *S. capitata* exposed to four treatments: control with ambient temperature and environmental CO_2_ [aTEaCO_2_], elevated CO_2_ at 600 ppm with ambient temperature [aTEeCO_2_], elevated temperature at +2 °C above ambient canopy temperature with environmental CO_2_ [eTEaCO_2_], and combined stress with elevated temperature (+2 °C above ambient canopy temperature) and elevated CO_2_ (600 ppm) [eTEeCO_2_]. Data are presented as mean ± SE, where ns indicates non-significant effects of any factor, F represents the ANOVA test statistic, and *p* denotes the significance level, with * *p* < 0.05. #: ANOVA analysis performed with Box–Cox transformed data. Letters are the Tukey mean comparison (*p* < 0.05).

Population	Source of Variation	A#	Ae #	Ho #	He	*Fis* #
Mothers	aTEaCO_2_	6.14 ± 0.70	4.72 ± 0.69	0.36 ± 0.11	0.77 ± 0.02	0.55 ± 0.13
Mothers	aTEeCO_2_	5.29 ± 0.86	3.84 ± 0.68	0.34 ± 0.14	0.70 ± 0.04	0.56 ± 0.17
Mothers	eTEaCO_2_	4.57 ± 0.75	3.09 ± 0.59	0.40 ± 0.12	0.62 ± 0.05	0.37 ± 0.19
Mothers	eTEeCO_2_	5.57 ± 0.61	3.59 ± 0.45	0.34 ± 0.09	0.70 ± 0.02	0.52 ± 0,12
	Mean	5.39 ± 0.36	3.81 ± 0.31	0.36 ± 0.06	0.70 ± 0.02	0.50 ± 0.07
Progeny	aTEaCO_2_	6.57 ± 0.61	4.60 ± 0.73 a	0.30 ± 0.12	0.76 ± 0.02 a	0.63 ± 0.13
Progeny	aTEeCO_2_	6.43 ± 0.80	3.91 ± 0.63 a	0.35 ± 0.13	0.72 ± 0.03 ab	0.54 ± 0.16
Progeny	eTEaCO_2_	5.57 ± 0.90	2.99 ± 0.57 a	0.28 ± 0.12	0.61 ± 0.05 b	0.59 ± 0.15
Progeny	eTEeCO_2_	6.29 ± 0.61	3.43 ± 0.50 a	0.30 ± 0.10	0.68 ± 0.03 ab	0.58 ± 0.11
	Mean	6.21 ± 0.35	3.73 ± 0.31	0.31 ± 0.06	0.69 ± 0.02	0.58 ± 0.07
ANOVA						
Progeny	aTEeCO_2_	F = 0.32, *p* = 0.57 ns	F = 0.00, *p* = 0.98 ns	F = 0.18, *p* = 0.67 ns	F = 0.14, *p* = 0.71 ns	F = 0.72, *p* = 0.41 ns
Progeny	eTEaCO_2_	F = 1.04, *p* = 0.32	F = 4.46, *p* = 0.045 *	F = 0.001, *p* = 0.97 ns	F = 6.30, *p* = 0.019 *	F = 0.11, *p* = 0.75 ns
Progeny	eTEeCO_2_	F = 0.76, *p* = 0.39 ns	F = 1.44, *p* = 0.24 ns	F = 0.27, *p* = 0.61 ns	F = 2.50, *p* = 0.13 ns	F = 0.44, *p* = 0.51 ns

**Table 5:** Analysis of Molecular Variance (AMOVA). Est. Var = estimated variance.

	Among Pops		Within Pops		Total		*p*-value
	Est. Var	(%)	Est. Var	(%)	Est. Var	(%)	
Mothers	0.061	1	8.394	99	8.456	100	0.304
Progeny	0.784	9	8.202	91	8.987	100	0.001
Mothers + Progeny	0.704	8	8.213	92	8.917	100	0.001

**Table 6:** *Fst* and Nm Analysis.

	Matrices		Progenies	
	Value	SE	Value	SE
*Fst*	0.066	0.005	0.065	0.007
Nm	3.713	0.363	3.843	0.449

## Data Availability

The data presented in this study are available on request from the corresponding author due to privacy concerns.

## Supplementary Materials

The following supporting information can be downloaded at: https://www.mdpi.com/article/10.3390/genes16080913/s1, File S1: Table S1. Seven microsatellite loci used for the amplification of *Stylosanthes capitata*, developed by (Santos-Garcia et al., 2011 [43]) and validated by (Alzate-Marin et al., 2019 [44]); File S1: Table S2. Average germination speed index (GSI), germination time (GT), and germination percentage (Gr-%) per week by treatment; File S1: Table S3. Allele frequencies in maternal and progeny populations (C = aTEaCO_2_, eC = aTEeCO_2_, eT = eTEaCO_2_, eTeC = eTEeCO_2_); File S1: Table S4. Analysis of mean values of genetic diversity parameters (Aa, Ae, Na, Ne, Ho, He, and F) between Maternal (M) and progeny (P) populations of *S. capitata* across treatments using the Mann–Whitney Test; File S1: Table S5. Analysis of Hardy–Weinberg equilibrium in maternal and progeny populations; File S1: Table S6. Pairwise *Fst* analysis between maternal and progeny populations. File S2: Table S1. Two-way ANOVA—Number of Seeds per Inflorescence—SPI. Analysis based on 40 observations; File S2: Table S2. Two-way ANOVA—100-Seed Weight (HSW). Analysis based on 64 observations; File S2: Table S3. Two-way ANOVA—Seed Abortions (%). Analysis based on 64 observations; File S2: Table S4. Two-way ANOVA—Non-viable seeds. Analysis based on 64 observations; File S2: Table S5. Three-way ANOVA—Seed Coat Color (6 levels × 3 sample groups × 12 maternal plants per treatment); File S2: Table S6. Three-way ANOVA—germination speed index (GSI). Analysis based on 48 observations; File S2: Table S7. Three-way ANOVA—Germination Time (GT). Analysis based on 48 observations; File S2: Table S8. Three-way ANOVA—Germination percentage (Gr). Analysis based on 48 observations; File S2: Table S9. Two-way ANOVA. Abnormal seedlings. Analysis based on 48 observations; File S2: Table S10. Two-Way ANOVA—Genetic Diversity Traits in Progeny (7 Molecular Markers SSR). Analysis based on 28 observations.
