# Supplementary material for: Warming in the Maternal Environment Alters Seed Performance and Genetic Diversity of Stylosanthes capitata, a Tropical Legume Forage
Source: Genes (Basel). 2025 Jul 30;16(8):913. doi: 10.3390/genes16080913 (PMC12385992; doi:10.3390/genes16080913)
Supplement: Supplementary file 1 [file genes-16-00913-s001.zip › File S1.pdf]

File S1 - Table S1. Seven microsatellite loci used for the amplification of *Stylosanthes capitata*, developed by Santos-Garcia et al. (2011 [43]) and validated by Alzate-Marin et al. (2019 [44]).

Loci	GenBank accession	Forward and Reverse Primers	Fragment Size (bp)	Repeat Motif
SC 18-01 A2A	GU734805	5′ AGCAGCATAGGGAATAAAAT 3′<br>5′ CAAAGGCCTAATCAACTGTG 3′	232-238	$(TC)_5(AC)_5$
SC 18-01 E4	GU734807	5′ CGGCAACTGGGAAAAATAA 3′<br>5′ ATGGGTAATCACAAATCTTCAG 3′	300-310	$(CA)_3CT(CA)_5$
SC 18-01T F2	GU734812	5′ CTGACCCCACCTAATGAGAAA 3′<br>5′ AGCAAAACAAAACAAACAACACTA 3′	196-198	$(TG)_7$
SC 18-01T G9	GU734810	5′ TCCAGCTAAAGGGCAACACA 3′<br>5′ CCACCGCACACCAGAGATT 3′	242-245	$(GAA)_7$
SC 18-01T G12A	GU734809	5′ ATGCTGATTTTTGGCTCTTTT 3′<br>5′ CCCCTTTTGAACGGATTG 3′	240-260	$(ATGGTA)_5$
SC 18-02 E12	GU734814	5′ AGGGGAAGGGCAAATGGT 3′<br>5′ GCATAGATGGCAAACAGAGACA 3′	270-305	$(GT)_5TA(GT)_3$<br>$(GA)_5CG(CA)_3$<br>$(CT)_3CA(CT)_{16}$
SC 18-01 H5	GU734808	5′ GCATCATTTGCATTTGTTTT 3′<br>5′ CTATCACCTCTCCATACCTTATC 3′	192-196	(TG)9

File S1 - Table S2. Average germination speed index (GSI), germination time (GT), and germination percentage (Gr - %) per week by treatment.

	GSI			GT			Gr (%)		
	32 week	40 week	71 week	32 week	40 week	71 week	32 week	40 week	71 week
$aTEaCO_2$	28.06±5	61.32±4.4	72.81±3.8	3.80±0.18	2.08±0.15	2.30±0.26	84±8	94.75±2	77.75±4
$aTEeCO_2$	33.26±3	78.44±1.3	50.56±8.0	3.80±0.11	1.98±0.37	1.70±0.04	93.5±3	96.75±0	84.5±4
$eTEaCO_2$	43.38±5.2	84.2±3.14	72.81±3.83	3.13±0.20	1.68±0.27	1.37±0.05	92.5±3	98±1	94.75±2
$eTEeCO_2$	37.28±2.6	69.12±7.9	70.69±2.3	3.51±0.26	2.02±0.12	1.86±0.18	88.5±2	98.75±1	95.0±0
Mean	35.49	73.27	66.72	3.56	1.94	1.80	89.63	97.06	88.00
SE	3.24<br>***	5.1<br>***	5.41<br>**	0.16	0.09<br>***	0.19<br>****	2.16	0.87	4.20
Tukey	b	a	a	a	b	b	a	a	a

File S1 - Table S3. Allele frequencies in maternal and progeny populations (C=aTEaCO_2, eC=aTEeCO_2, eT=eTEaCO_2, eTeC= eTEeCO_2).

Locus	Allele	Mothers (C)	Mothers (eC)	Mothers (eT)	Mothers (eTeC)	Progeny (C)	Progeny (eC)	Progeny (eT)	Progeny (eTeC)
A2A	228	0.05556	0.05	0	0.05	0.04577	0.05	0	0.03481
A2A	234	0.05556	0	0.05	0.05	0.06338	0.0625	0.05449	0.06646
A2A	238	0.11111	0.15	0.05	0.1	0.10915	0.14063	0.03526	0.08544
A2A	240	0.27778	0.35	0.65	0.4	0.30282	0.31563	0.66987	0.48418
A2A	242	0.27778	0.4	0.1	0.4	0.27817	0.38438	0.10897	0.32911
A2A	244	0.11111	0	0.05	0	0.09155	0	0.05449	0
A2A	246	0.11111	0.05	0	0	0.10915	0.04688	0	0
A2A	248	0	0	0.1	0	0	0	0.07692	0
E4	296	0.11111	0	0	0	0.11268	0	0	0.03125
E4	298	0	0	0	0.1	0	0	0	0.06875
E4	300	0.33333	0.3	0	0.2	0.34859	0.28808	0	0.2
E4	304	0.27778	0.6	0.45	0.5	0.32042	0.57947	0.47484	0.49688
E4	308	0.11111	0.1	0.4	0.15	0.09507	0.10927	0.39623	0.16875
E4	311	—	—	—	—	0	0.01325	0	0
E4	316	—	—	—	—	0	0	0	0.00313
E4	318	0.11111	0	0.15	0.05	0.08451	0.00993	0.12893	0.03125
E4	326	0.05556	0	0	0	0.03873	0	0	0
F2	190	0.11111	0.125	0	0.05	0.08394	0.15652	0	0.08176
F2	192	0.05556	0.125	0	0.2	0.05109	0.08696	0.00725	0.19497
F2	194	0.11111	0.0625	0.05556	0.1	0.08394	0.07826	0.05797	0.06918
F2	196	0.27778	0.25	0.16667	0	0.28467	0.2	0.17754	0.00629
F2	197	—	—	—	—	0	0	0.00362	0
F2	200	0.44444	0.4375	0.72222	0.55	0.4562	0.46957	0.71014	0.58805
F2	204	0	0	0.05556	0.05	0.04015	0.0087	0.03261	0.02201
F2	208	0	0	0	0.05	0	0	0.01087	0.03774
G9	200	0	0.1	0	0	0	0.11511	0	0
G9	240	0.35	0.3	0.44444	0.1	0.34314	0.25899	0.44097	0.10127
G9	242	0.15	0	0	0.1	0.13399	0.0036	0.00347	0.0981
G9	246	—	—	—	—	0.00327	0	0	0.00316
G9	248	0.2	0.5	0.55556	0.4	0.20588	0.47482	0.54861	0.4019
G9	250	0.25	0.1	0	0.3	0.28758	0.13309	0	0.33861
G9	258	0.05	0	0	0.1	0.02614	0.01439	0.00694	0.05696
G12A	254	0	0.0625	0	0	0	0.00813	0	0
G12A	260	0	0.0625	0.1	0.375	0.01724	0.10569	0.09539	0.37681
G12A	262	0.1	0	0	0	0.10345	0	0	0
G12A	266	0.35	0.375	0.15	0.375	0.27931	0.36585	0.16776	0.47101
G12A	270	0.1	0.1875	0.35	0.125	0.1069	0.19106	0.38816	0.13043
G12A	272	0.25	0.25	0.1	0.125	0.32414	0.25203	0.08553	0.02174
G12A	278	0.2	0.0625	0.3	0	0.16897	0.07724	0.26316	0

File S1 - Table S3. Continuation…

Locus	Allele	Mothers (C)	Mothers (eC)	Mothers (eT)	Mothers (eCeT)	Progeny (C)	Progeny (eC)	Progeny (eT)	Progeny (eTeC)
E12	288	0.1	0	0.05	0	0.11613	0	0.03289	0
E12	290	0	0	0.1	0.05	0	0	0.12171	0.05592
E12	292	0.05	0.11111	0.05	0.25	0.05484	0.10219	0.10526	0.22368
E12	296	0.1	0.05556	0.2	0.2	0.10645	0.05839	0.09868	0.17105
E12	298	0.1	0.05556	0.15	0.05	0.0871	0.04745	0.14474	0.05263
E12	302	0.15	0.22222	0	0	0.12258	0.23358	0.01974	0
E12	308	0.05	0.05556	0	0.15	0.05161	0.06204	0.01316	0.12829
E12	318	0.15	0.11111	0.1	0.05	0.15161	0.10584	0.07895	0.0625
E12	322	0.1	0.05556	0.25	0.15	0.10323	0.05109	0.30921	0.22039
E12	325	0.05	0.05556	0	0	0.04839	0.04745	0	0
E12	328	0.15	0.16667	0.1	0.05	0.15806	0.17883	0.07566	0.04605
E12	330	—	—	—	—	0	0.0073	0	0
E12	337	0	0.11111	0	0.05	0	0.10584	0	0.03947
H5	194	0	0.11111	0	0	0	0.11189	0	0
H5	198	0	0	0	0.05	0	0	0	0.0487
H5	200	0.4	0.33333	0.22222	0.35	0.40068	0.33217	0.22695	0.35065
H5	202	0.15	0.22222	0.5	0.4	0.18836	0.22028	0.53191	0.42208
H5	204	0.3	0	0.22222	0.1	0.32877	0.00699	0.21986	0.05195
H5	206	0.1	0.33333	0	0.1	0.07877	0.32867	0	0.08442
H5	208	0.05	0	0.05556	0	0.00342	0	0.02128	0.04221
Total Number	59	43	37	32	39	46	45	39	44

File S1 - Table S4. Analysis of mean values of genetic diversity parameters (Aa, Ae, Ho, He, and F) between Maternal (M) and progeny (P) populations of *Stylosanthes capitata* across treatments using the Mann-Whitney Test.

	Na (M)	Na (P)	Ne (M)	Ne (P)	Ho (M)	Ho (P)	He (M)	He (P)	F (M)	F (P)
$aTEaCO_2$										
Mean	6.14	6.57	4.72	4.58	0.36	0.30	0.77	0.76	0.55	0.63
SE	0.70	0.61	0.70	0.73	0.11	0.12	0.02	0.03	0.13	0.13
Z (*p-value*)	0.938 (0.35)		0.511 (0.61)		0.640 (0.52)		0.511 (0.61)		0.766 (0.44)	
$aTEeCO_2$										
Mean	5.29	6.43	3.84	3.92	0.34	0.36	0.70	0.72	0.56	0.54
SE	0.87	0.78	0.68	0.63	0.14	0.13	0.04	0.03	0.17	0.16
Z (*p-value*)	1.588 (0.11)		0.255 (0.80)		0.322 (0.75)		0.255 (0.80)		0.513 (0.61)	
$eTEaCO_2$										
Mean	4.57	5.57	3.09	2.99	0.40	0.28	0.62	0.61	0.37	0.59
SE	0.75	0.90	0.59	0.56	0.13	0.13	0.05	0.05	0.19	0.15
Z (*p-value*)	0.713 (0.48)		0.127 (0.90)		0.960 (0.34)		0.127 (0.90)		0.511 (0.61)	
$eTEeCO_2$										
Mean	5.57	6.29	3.59	3.42	0.34	0.30	0.70	0.68	0.52	0.58
SE	0.61	0.61	0.45	0.50	0.09	0.10	0.02	0.03	0.12	0.11
Z (*p-value*)	1.054 (0.29)		0.638 (0.52)		0.127 (0.90)		0.638 (0.52)		0.127 (0.90)	

File S1 - Table S5. Analysis of Hardy–Weinberg equilibrium in maternal and progeny populations.

Maternal population (n=40)							
Loci	**Ho**	**He**	**He-Ho**	***Fis***	**HWE Test**		
					χ^2	**GL**	**p**
A2A	0.356	0.734	0.378	0.475	53.63	8	<0.001
E4	0.150	0.718	0.568	0.761	96.62	8	<0.001
G9	0.100	0.720	0.620	0.846	107.15	8	<0.001
F2	0.475	0.672	0.197	0.238	15.66	8	<0.001
G12	0.400	0.824	0.424	0.455	31.66	8	<0.001
E12	0.950	0.903	-0.047	-0.106	127.53	8	<0.001
H5	0.078	0.771	0.693	0.888	127.53	8	<0.001
Progeny population (n=640)							
Locus	**Ho**	**He**	**He-Ho**	***Fis***	**HWE Test**		
					χ^2	**GL**	**p**
A2A	0.303	0.675	0.372	0.548	127.54	8	<0.001
E4	0.099	0.652	0.553	0.848	127.54	8	<0.001
G9	0.089	0.659	0.570	0.864	127.54	8	<0.001
F2	0.325	0.617	0.292	0.470	127.54	8	<0.001
G12	0.305	0.722	0.417	0.574	127.54	8	<0.001
E12	0.954	0.860	-0.094	-0.113	127.54	8	<0.001
H5	0.076	0.682	0.606	0.887	127.54	8	<0.001

File S1 - Table S6. Pairwise *Fst* analysis between maternal and progeny populations.

Maternal				Progeny				
aTEaCO_2	aTEeCO_2	eTEaCO_2	eTEeCO_2	aTEaCO_2	aTEeCO_2	eTEaCO_2	eTEeCO_2	
0.000								aTEaCO_2
0.028	0.000							aTEeCO_2
0.058	**0.060**	0.000						eTEaCO_2
0.038	0.030	0.055	0.000					eTEeCO_2
0.001	0.028	0.057	0.037	0.000				aTEaCO_2
0.026	0.001	0.058	0.026	0.026	0.000			aTEeCO_2
0.061	0.060	0.001	0.054	**0.059**	0.058	0.000		eTEaCO_2
0.043	0.035	0.053	0.003	0.043	0.031	0.052	0.000	eTEeCO_2
