# Supplementary material for: Warming in the Maternal Environment Alters Seed Performance and Genetic Diversity of Stylosanthes capitata, a Tropical Legume Forage
Source: Genes (Basel). 2025 Jul 30;16(8):913. doi: 10.3390/genes16080913 (PMC12385992; doi:10.3390/genes16080913)
Supplement: Supplementary file 1 [file genes-16-00913-s001.zip › File S2.pdf]

File S2 - Table S1. Two-way ANOVA – Number of Seeds per Inflorescence -SPI. Analysis based on 40 observations.

**Source of Variation**	**SS**	**df**	**MS**	**F-value**	**p-value (1,36)**	**η² (Effect Size)**	**Interpretation**
Temperature	232.324	1	232.324	12.80	**0.0010**	**0.256**	Highly significant (**)
CO_2	3.249	1	3.249	0.179	0.6747	0.0036	Not significant
Interaction	19.044	1	19.044	1.049	0.3125	0.0210	Not significant
Residual (Error)	653.254	36	18.146	—	—	—	—
Total N	—	40	—	—	—	—	—
Total SS	907.871	39	—	—	—	—	—

File S2 - Table S2. Two-way ANOVA – 100-Seed Weight (HSW). Analysis based on 64 observations.

**Source of variation**	**SS**	**df**	**MS**	**F-value (1,60)**	**p-value**	**η² (Effect size)**	**Interpretation**
Temperature	0.00090	1	0.00090	1.144	0.2891	0.0187	Not significant
CO_2	6.25E-06	1	6.25E-06	0.0079	0.9293	0.00013	Not significant
Interaction	5.63E-05	1	5.63E-05	0.0715	0.7901	0.00117	Not significant
Residual (error)	0.04721	60	0.0007869	—	—	—	—
Total N	—	64	—	—	—	—	—
Total SS	0.04817255						

File S2 - Table S3. Two-way ANOVA – Seed Abortions (%). Analysis based on 64 observations.

**Source of variation**	**SS**	**df**	**MS**	**F-value (1,60)**	**p-value**	**η² (Effect size)**	**Interpretation**
Temperature	0.06338	1	0.06338	6.517	**0.01325**	**0.0964**	Significant (*)
CO_2	0.00032	1	0.00032	0.03291	0.8566	0.00049	Not significant
Interaction	0.01033	1	0.01033	1.062	0.3068	0.0157	Not significant
Residual (error)	0.58352	60	0.00973	—	—	—	—
Total N	—	64	—	—	—	—	—
Total SS	0.65755						

File S2 - Table S4. Two-way ANOVA – Non-viable seeds. Analysis based on 64 observations.

**Source of variation**	**SS**	**df**	**MS**	**F-value (1,60)**	**p-value**	**η^2 (Effect size)**	**Interpretation**
Temperature	0.12721	1	0.12721	8.698	**0.00453**	**0.123**	Highly significant (**)
CO_2	0.00141	1	0.00141	0.0965	0.7571	0.0013	Not significant
Interaction	0.02993	1	0.02993	2.047	0.1577	0.0288	Not significant
Residual (error)	0.87750	60	0.01462	—	—	—	
Total N	—	64	—	—	—	—	—
Total SS	1.03609						

File S2 - Table S5. Three-way ANOVA – Seed Coat Color (6 levels × 3 sample groups × 12 maternal plants per treatment).

**Source of Variation**	**SS**	**df**	**MS**	**F-value (df_1, df_2)**	**p-value**	**η^2(Effect size)**	**Interpretation**
Seed Coat Color	3.894	5	0.7788	63.36 (5, 48)	**<0.0001**	**0.7360**	Highly significant (***)
Temperature	0.05134	1	0.05134	4.177 (1, 48)	**0.0465**	**0.0097**	Significant (*)
CO_2	1.49E-06	1	1.49E-06	0.0001 (1, 48)	0.9913	0.0000003	Not significant
Seed Coat × Temperature	0.7312	5	0.1462	11.90 (5, 48)	**<0.0001**	**0.1382**	Highly significant (***)
Seed Coat × CO_2	0.01416	5	0.00283	0.23 (5, 48)	0.9474	0.0027	Not significant
Temperature × CO_2	0.00053	1	0.00053	0.043 (1, 48)	0.8364	0.0001	Not significant
Seed Coat × Temp × CO_2	0.01053	5	0.00211	0.171(5, 48)	0.9720	0.0020	Not significant
Residual (error)	0.5900	48	0.01229	—	—	—	—
Total SS	5.292	—	—	—	—	—	—

File S2 - Table S6. Three-way ANOVA – Germination Speed Index (GSI). Analysis based on 48 observations.

Source of Variation	SS	df	MS	F-value (df_1, df_2)	p-value	η^2(Effect size)	Interpretation
Week	13041	2	6520.5	75.60 (2,36)	**<0.0001**	**0.6764**	Strong temporal influence(***)
Temperature	937	1	937.0	10.90 (1,36)	**0.0022**	**0.0486**	Highly significant (**)
CO_2	180	1	180.0	2.08 (1,36)	0.1575	0.0093	Not significant
Temperature × CO_2	182	1	182.0	2.11 (1,36)	0.1548	0.0094	Not significant
Week × Temperature	25.7	2	12.85	0.149 (2,36)	0.8621	0.0013	Not significant
Week × CO_2	419	2	209.5	2.43 (2,36)	0.1026	0.0217	Not significant
Week × Temperature × CO_2	1388	2	694.0	8.05 (2,36)	**0.0013**	**0.0720**	Highly significant (**)
Residual	3105	36	86.25	—	—	—	—
Total SS	19277.7						

File S2 - Table S7. Three-way ANOVA – Germination Time (GT). Analysis based on 48 observations.

Source of Variation	SS	df	MS	F-value (df_1, df_2)	p-value	η^2 (Effect size)	Interpretation
Week	30.71	2	15.36	92.20(2, 36)	**<0.0001**	**0.765**	Strong temporal influence (***)
Temperature	1.483	1	1.483	8.904(1, 36)	**0.0051**	**0.036**	Highly significant(**)
CO_2	0.1164	1	0.1164	0.6986 (1, 36)	0.4088	0.003	Not significant
Temperature x CO_2	1.124	1	1.124	6.748 (1, 36)	**0.0135**	**0.028**	Significant
Week x Temperature	0.1875	2	0.0937	0.5629 (2, 36)	0.5745	0.005	Not significant
Week x CO_2	0.163	2	0.0815	0.4895 (2, 36)	0.6170	0.004	Not significant
Week x Temperature x CO_2	0.3395	2	0.1698	1.019 (2, 36)	0.3710	0.008	Not significant
Residual	5.996	36	0.1666	—	—	—	—
Total SS	40.1194						

File S2 - Table S8. Three-way ANOVA – Germination percentage (Gr). Analysis based on 48 observations.

Source of Variation	SS	df	MS	F-value (df_1, df_2)	p-value	η^2 (Effect size)	Interpretation
Week	0.2867	2	0.1434	10.92 (2, 36)	**0.0002**	**0.263**	Significant temporal influence(***)
Temperature	0.1555	1	0.1555	11.85 (1, 36)	**0.0015**	**0.143**	Highly significant(**)
eCO_2	0.00900	1	0.0090	0.6860 (1, 36)	0.413	0.008	Not significant
Week × Temperature	0.08631	2	0.0431	3.288 (2, 36)	0.0488	0.079	Significant(*)
Week × CO_2	0.00049	2	0.0002	0.0190 (2, 36)	0.9812	0.0005	Not significant
Temperature × CO_2	0.04676	1	0.0467	3.562 (1, 36)	0.0672	0.043	Not significant
Week × Temperature × CO_2	0.02898	2	0.0144	1.104 (2, 36)	0.3425	0.026	Not significant
Residual (Erro)	0.4725	36	0.0131	—	—	—	—
Total SS	1.08625						

File S2 - Table S9. Two-way ANOVA. Abnormal seedlings. Analysis based on 48 observations.

Source of variation	SS	df	MS	F-value (1,44)	p-value	η^2 (Effect size)	Interpretation
Temperature	0.06386	1	0.06386	8.818	**0.0048**	**0.1653**	Highly significant (**)
CO_2	0.003584	1	0.003584	0.4948	0.4855	0.0093	Not significant
Temperature × CO_2	0.0001807	1	0.0001807	0.0249	0.8752	0.0005	Not significant
Residual (error)	0.3187	44	0.007242	—	—	—	—
Total N	—	48	—	—	—	—	—
Total SS	0.386324						

File S2 - Table S10. Two-Way ANOVA —Genetic Diversity Traits in Progeny (7 Molecular Markers SSR). Analysis based on 28 observations.

Parameter	Source of variation	SS	df	MS	F (df_1, df_2)	p-value	η^2 (Effect size)	Interpretation
**A**	Temperature	0.1094	1	0.1094	1.044 (1, 24)	0.3171	0.0399	Not significant
	CO_2	0.0339	1	0.0339	0.324 (1, 24)	0.5746	0.0124	Not significant
	Interaction	0.0799	1	0.0799	0.763 (1, 24)	0.3912	0.0292	Not significant
	Residual (error)	2.515	24	0.1048	—	—	—	
**Ae**	Temperature	0.6646	1	0.6646	4.465 (1, 24)	**0.0452**	**0.1493**	Significant (*)
	CO_2	0.00008	1	0.00008	0.0005 (1,24)	0.9818	0.00002	Not significant
	Interaction	0.2139	1	0.2139	1.437 (1, 24)	0.2423	0.0480	Not significant
	Residual (error)	3.61	24	0.1524				
**Ho**	Temperature	0.0015	1	0.0015	0.0014 (1, 24)	0.9701	0.00006	Not significant
	CO_2	0.2038	1	0.2038	0.1887 (1, 24)	0.6679	0.0077	Not significant
	Interaction	0.2927	1	0.2927	0.2710 (1, 24)	0.6074	0.0111	Not significant
	Residual (error)	25.92	24	1.08	—	—	—	
**He**	Temperature	0.0573	1	0.0573	6.296 (1, 24)	**0.0193**	**0.1912**	Significant (*)
	CO_2	0.0012	1	0.0012	0.137 (1, 24)	0.7144	0.0042	Not significant
	Interaction	0.0228	1	0.0228	2.504 (1, 24)	0.1267	0.0760	Not significant
	Residual (error)	0.2185	24	0.009106	—	—	—	
Fis	Temperature	0.0119	1	0.0119	0.1073 (1, 20)	0.7467	0.0050	Not significant
	CO_2	0.0794	1	0.0794	0.7153 (1, 20)	0.4077	0.0336	Not significant
	Interaction	0.0490	1	0.0490	0.4421 (1, 20)	0.5137	0.0208	Not significant
	Residual (error)	2.219	20	0.1109	—	—	—	
